# Intra-Abdominal Hemorrhagic Catastrophe due to Large Subserous Myomatous Capsular Venous Rupture

**DOI:** 10.1155/2022/2696213

**Published:** 2022-01-04

**Authors:** Tamilselvi Sethupathy, Madhankumar Madathupalayam, Krithika Arun Prasad

**Affiliations:** ^1^Department of Obstetrics and Gynaecology, CK Medical Centre Hospital, 41, Balasubbarayalu Street, Erode, 638001 Tamil Nadu, India; ^2^CK Medical Centre Hospital, 41, Balasubbarayalu Street, Erode, 638001 Tamil Nadu, India

## Abstract

Uterine leiomyoma is a common benign uterine tumor of women in the reproductive age group. Although the common symptoms of leiomyoma are menorrhagia and dysmenorrhoea, this patient presenting as a near collapse is a rare finding. The patient presented with abdominal pain and worsening anemia within a span of hours and hypotension, tachycardia, and tachypnoea needing urgent surgical intervention and blood transfusion and intensive care support is relatively rare. Though every effort to know the exact cause of intraperitoneal hemorrhage in this patient was taken, the rarer diagnosis of capsular venous rupture was not identified prior to surgical intervention. Initially, laparoscopy was introduced first to identify the cause of massive hemorrhage; the approach was changed to open myomectomy keeping in mind the general condition of the patient. Hence, for any patient with a prior diagnosis of myoma with hemodynamic instability, the rarer diagnosis of leiomyomatous capsular venous erosion should be a differential diagnosis to aid in the appropriate management of the women. The team of interdisciplinary expertise will definitely improve the outcomes in such cases.

## 1. Introduction

Uterine leiomyomas are the most prevalent benign tumors of women in the reproductive age group arising from the proliferation of myometrial cells [1] The incidence of fibroids is highest in women of African origin who tend to have larger fibroids which tend to be multiple and more symptomatic at the time of presentation [2]; this complication of hemodynamic instability due to capsular venous rupture causing massive intra-abdominal hemorrhage in our patient of Asian origin is rare [3]. Intra-abdominal hemorrhage due to uterine fibroids is rare as evidenced by the identification of only 125 cases in the English language literature since 1902 [4]. The source of hemorrhage in over half of the patients identified was a ruptured superficial vessel overlying a leiomyoma [5, 6] or could be posttraumatic [7, 8]. There is a historical scientific basis supporting abnormal vasculature of subserosal fibroids that is susceptible to spontaneous rupture [9]. The case is reported for the rarity of presentation of a common tumor and the dilemmas of the clinician in the diagnosis and to stress the need for an interdisciplinary team in improving the outcome.

## 2. Patient Information

**Figure 1:**
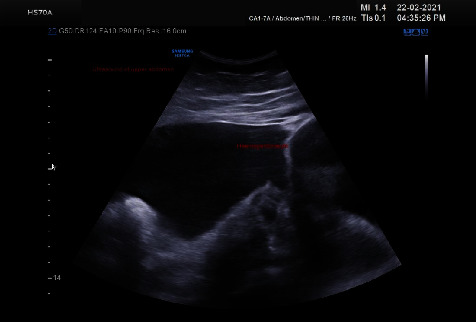
Ultrasound of the upper abdomen showing massive hemoperitoneum.

**Figure 2:**
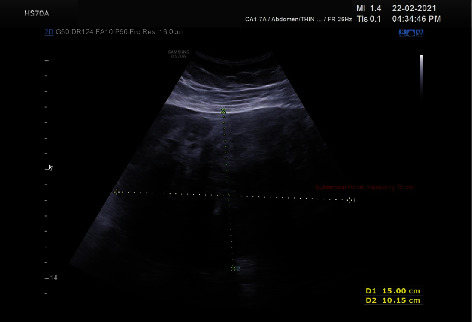
Ultrasound of the lower abdomen showing fibroid measuring about 15 cm.

This patient is aged 34 years, P2 L2 with previous 2 caesarean sections with last child birth 6 years back and had the last menstrual period 21 days back. Her cycles were regular and no prior history of dysmenorrhoea. Bowel and bladder habits were normal. The patient developed mild abdominal pain a day prior. She sought consultation with her primary consultant as her pain worsened since morning. On admission, the patient was evaluated for anemia and her hemoglobin was 8.0 gms%. She was administered pain reliever, and her abdominal palpation revealed a firm mass in the lower abdomen and mild tenderness in the lower abdomen. As then, an ultrasound was done. Ultrasound revealed echogenic free fluid suggestive of intraperitoneal hemorrhage in the abdomen. A myoma of size 15 × 10 cm, 5.9 × 4.7 cm, was seen with an endometrial thickness of 8 mm. Bilateral ovaries appeared polycystic (Figures 1 and 2).

Meanwhile, attempts were made to rule out other common causes of intraperitoneal hemorrhage such as ectopic pregnancy and corpus luteal rupture. Urinary *β-*HCG was 1.2 mIu/L. The patient was advised for CT as the cause of hemoperitoneum could not be established. The patient then complained of dyspnoea on lying down and severe abdominal discomfort. Hence, a preoperative diagnosis of intra-abdominal hemorrhage of unknown origin was established with the incidental finding of a uterine fibroid as what happened in 96% of similar cases reported in literature [4].

On examination, the patient was tachypnoeic and had tachycardia and blood pressure 90/60 mmHg. Meanwhile, packed cell transfusion started and the consent for surgical intervention was obtained. A team of gynecologist, general surgeon, and anesthetist formed as the definitive diagnosis was not established as outlined in this study [4]. Under general anesthesia, laparoscopy was done. Massive hemoperitoneum with blood clots covering the whole uterine surface was seen extending up to the upper abdomen. The collected blood suctioned was about 3000 mL (Video 1).

Fibroids subserous in location, about 15 cm and 5 cm, were seen. No evidence of torsion or evidence of degeneration was seen. The uterus was seen to be of normal size. As the collected blood was removed, a gush of blood in the cleft between the fibroids was seen. Venous rupture of the capsule between the two subserous myomas was found. However, as the bleeding from the venous end was profuse, we decided for open myomectomy (Video 2).

The base of the myoma was fundal in location, and myomectomy was done. Ovaries are found to be normal. The abdomen closed in layers. The patient was transfused with six units of packed cells and 4 units of fresh frozen plasma.

**Figure 3:**
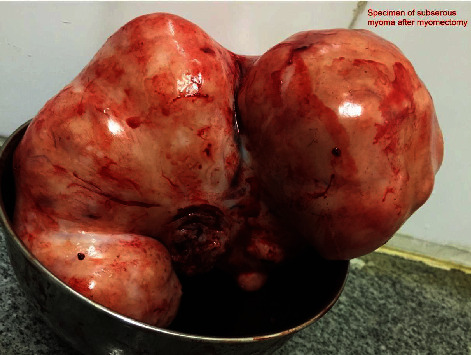
Subserous fibroid after myomectomy.

**Figure 4:**
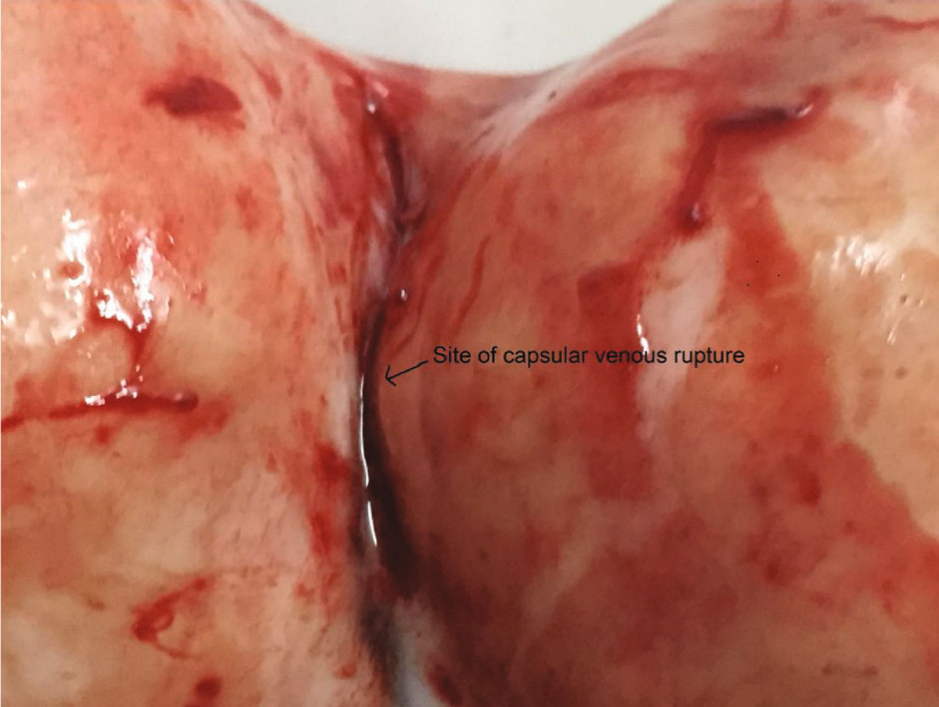
Site of capsular venous rupture in the subserous fibroid postmyomectomy.

Post-op period was uneventful, and the patient was discharged on the 4th postoperative day. The histopathological diagnosis was benign leiomyoma with no evidence of degeneration (Figures 3 and 4).

## 3. Discussion

The causes of hemoperitoneum in connection with leiomyoma include bleeding from a subserosal artery [6], rupture of a subserosal vein overlying a uterine myoma [5], an avulsed pedunculated leiomyoma [7], a ruptured leiomyoma [8, 10], or a lacerated leiomyoma [11]. In most cases, bleeding from a uterine leiomyoma has been associated with trauma or torsion of the tumor. The most common cause of hemoperitoneum in patients with large myoma was the rupture of superficial blood vessels over the surface of a fibroid, identified in 60.8% of women; all of whom were diagnosed only intraoperatively as in our patient [4]. Among these cases, 76.3% are venous in origin, while 11.3% were arterial, which is still more rare [6]. Increased abdominal pressure due to hard work, defecation, sports, violent coitus, pregnancy, and menstruation are predisposing factors for rupture of the superficial vessels on uterine leiomyoma although some patients have no such aggravating factors [11].

The cause of hemorrhage in this case is venous in origin, and there are no predisposing factors identified for rupture. It has been postulated that a myoma larger than 10 cm can overstretch the covering vessel, resulting in the rupture of the feeding vessel, and two that the myomas can enlarge and split the feeding vessels between the uterine masses [11]. Congestion of a vein overlying a leiomyoma, irrespective of the patient's age or parity or size of the leiomyoma, is a risk factor for rupture [12]. The abnormal pattern of vasculature in fibroids, if subserosal in location, was established by Sampson in his studies [9]. Even though this condition is extremely rare, every clinician has to bear in mind that acute fibroid complications can be a potential cause of acute abdominal pain and massive hemorrhage that requires immediate surgery [4].

Abdominal pain was the most common symptom described on initial presentation with worsening pain since then. About 83.2% presented with sudden onset lower abdominal pain while 16% experienced a gradual onset over 3–5 days [5]. Women presenting with cardiovascular collapse [13] have been documented, and there are reports of severe morbidity and mortality due to late presentation or delay in establishing the diagnosis as the mortality is about 3.2% [4].

Establishing a diagnosis in cases of large pelvic tumors with hemoperitoneum is difficult [14]. In both pregnant and nonpregnant patients presenting with abdominal pain and concern for active hemorrhage, point-of-care ultrasound (POCUS) has been utilized for triage and management decision-making [15]. The ultrasound aided in diagnosis of intra-abdominal hemorrhage and the large size of fibroid in our case. Although CT was planned as the extravasation of contrast material could have given a clue to diagnosis, the hemodynamic status of the patient precluded further investigation. MRI has limited availability in the emergency department, and it is not currently included in the American College of Radiology Appropriateness Criteria.

The differential diagnoses are twisted adnexa, ruptured ectopic pregnancy, hemorrhagic corpus luteum, or follicular cyst, while that of the pelvic mass would be ovarian or endometrial carcinoma, uterine sarcoma or leiomyoma, and, rarely, ovarian fibroma [3]. Various differential diagnoses have been entertained and necessary steps taken to rule out the cause of massive bleeding. The commonest cause of massive hemorrhage in women of reproductive age group is ectopic pregnancy. The urine pregnancy test and serum titers of beta human chorionic gonadotropin levels ruled out the possible diagnosis. The other diagnosis was corpus luteal cyst, although the large hemorrhage and visualization of normal ovaries ruled out the cause [16]. Other surgical causes of spontaneous intra-abdominal hemorrhage include rupture of superior mesenteric, middle colic, and coeliac arteries and aorta which is common in male and often associated with hypertension [17]. Even minor strains like vomiting or purging may cause rupture of atherosclerotic or aneurysmal vessels although such history is denied by the patient. Other rare causes of splenic rupture accompanying the incidental finding of myoma should be considered [18].

The definitive treatment of myoma is myomectomy or sometimes hysterectomy either by laparoscopy, hysteroscopy, or laparotomy in tumors of large size [19]. Laparotomy was the most common surgical approach, with laparoscopy successfully performed in only very few cases (1.6%) [4] in such catastrophic situations. Laparoscopic surgery was attempted in some cases of which most were converted to open as in our case, mainly due to uncertainty in the diagnosis. The postoperative complication was nil, and the histopathological confirmation of the benign nature of the tumor was established with no evidence of degeneration.

## 4. Takeaway Points

1. The clinicians should be aware of the complications to aid in timely diagnosis and management of such rare catastrophes
2. The interdisciplinary team of gynecologist, anesthetist, and surgeons would help to tackle cases of uncertain diagnosis

## 5. Conclusion

Further research regarding the cause and predisposing factors of such massive hemorrhage in subserous myoma would be life-saving as timely management is rewarding.

## Supplementary Materials

Supplementary 1 Video 1 Laparoscopic video showing the massive hemoperitoneum with clotted blood covering the entire uterus and the large subserous myoma further obscuring the site of bleeding.

Supplementary 2 Video 2 Laparoscopic video showing ruptured actively bleeding vein on the surface of the capsule of subserous myoma.
